# Analysis of rhizosphere fungal diversity in lavender at different planting years based on high-throughput sequencing technology

**DOI:** 10.1371/journal.pone.0310929

**Published:** 2024-10-03

**Authors:** Xia Deng, Renzeng Shi, Rehab O. Elnour, Zixuan Guo, Junzhu Wang, Wenwen Liu, Guihua Li, Ziwei Jiao

**Affiliations:** 1 College of Biological Science and Technology, Yili Normal University, Yining, Xin Jiang, China; 2 Xinjiang Key Laboratory of Lavender Conservation and Utilization at Yili Normal University, Yining, Xin Jiang, China; 3 Faculty of Sciences and Arts, Biology Department, King Khalid University, Dahran Al-Janoub, Saudi Arabia; Sakarya Uygulamali Bilimler Universitesi, TÜRKIYE

## Abstract

Continuous cropping is a common cultivation practice in lavender cultivation, and the structure of the soil microbial community is one of the main reasons affecting the continuous cropping disorder in lavender; however, the relationship between the number of years of cultivation and inter-root microbial composition has not yet been investigated; using Illumina high-throughput sequencing we detected fungal community structure of rhizosphere soil under 1 (L1), 3 (L3), 5 (L5) and 0 (L0) years’ of lavender cultivation in Yili, Xinjiang China. The results showed that with the extension of planting years, the physical-chemical characteristics of the soil shifted, and the diversity of the fungal communities shrank, the abundance and richness of species decreased and then increased, and the phylogenetic diversity increased, The structure of the soil fungal communities varied greatly. At phylum level, dominant fungal phyla were *Ascomycetes*, *Basidiomycetes*, etc. At genus level, dominant genera were *Gibberella*, *Mortierella*, etc, whose absolute abundance all increased with increasing planting years (*P* < 0.05); redundancy analysis showed that thesoil physicochemical characteristics significantly correlated with dominant bacterial genera. The FUN Guild prediction showed that six groups of plant pathogens and plant saprotrophs changed significantly (*P <* 0.05), the amount of harmful bacteria in the soil increased while the amount of arbuscular mycorrhizal fungui (AMF) decreased, leading to a continuous cropping obstacle of lavender. The findings of this study provida theoretical foundation for the management of continuous cropping and the prevention fungus-related diseases in lavender.

## Introduction

The rhizosphere is home to a wide range of microorganisms, and their suitability to the host plants stabilizes the ecosystem. In long-term cultivation, planting years affect the composition of the rhizosphere microbial community [1], leading to an imbalance in the rhizosphere microecosystem [2–6]. With the development of molecular biotechnology such as high throughput sequencing and its application in combination with traditional methods in recent years, researchers have increasingly studied plant-associated microorganisms and their drivers [7–9]. more research has been done on the relationship between soil microbial diversity and its roles in natural ecosystems. Studies based on *Arabidopsis thaliana*, rice, maize, wheatand other plants have also revealed that the plant growth cycle controls the ecological processes of rhizosphere microbial communities [10–13].

Lavender *(Lavandula angustifolia* Mill*)* belongs to the family Labiatae, a perennial subshrub, which likes warmth and light, is a class of ornamental, edible, medicinal, greening and can be deep-processed and other wide range of uses of urban agriculture cultivars [14]. Yili, Xinjiang is China’s largest lavender planting base, known as the world’s three major origins of lavender planting areas with Provence, France and Hokkaido, Japan. In Yili, continuous planting for 8–15 years has led to frequent outbreaks of pests and diseases, further reducing production [15,16]. In recent years, pests and diseases of lavender have been reported in China, France, Bulgaria and the United States [17–21]. The lifespan of l Lavender has been reduced from over 10 years of cultivation to 3 years [22]. The structure of the rhizosphere microbial community structures is affected by continuous monoculture; soil phosphorus, nitrogen, potassium, pH and organic matter decrease and soil acidification occurs [23]. This disrupts the proliferation of rhizosphere microbes and leads to a reduction in beneficial microbes and an increase in harmful microbes [24–27]. Although standardized production of lavender has been achieved in Yili, research on nutrient management lavender growth is still lacking, focusing mainly on crop cultivation techniques, essential oil extraction, and pathology, However, little research has been reported on the diversity of rhizosphere fungi in the lavender succession [28]. Fungal plant pathogens are important threats to soil and plant health, but the mechanism of their effect on lavender needs to be further analyzed.

Therefore, in this study, high-throughput sequencing technology was applied assess the changes in the inter-root fungal community of lavender due to the number of years of continuous cropping, and we hypothesized that (a) continuous cropping would cause significant differences in the inter-root fungal community of lavender, and (b) the increase in the number of years of cropping of inter-root fungal taxa would cause changes in soil fungal function which in turn would cause fungal diseases in lavender. In this study, we used high-throughput sequencing to study the inter-root fungi of lavender in four planting years, to examine the shift in fungal community diversity and the changing pattern of pathogens, hopefully providing a theoretical foundation for systematic planting of lavender as well as the prevention and control of continuous cropping barriers.

## Materials and methods

### Study area and soil sampling

The study area is located in Tianshan Flower Sea (43°71′ N, 81°99′ E), Yining County, Xinjiang. It has a low annual rainfall of about 340 mm and a temperate continental climate, with an annual mean temperature of 9°C, total solar radiation of 134.5 k kcal/cm^2^, and annual evaporation of about 1,621 mm. The lavender cultivar was New Kaon 2 Space Blue, transplanted in seedlings, with row spacing of about 120 cm and drip irrigation, and its fertilization, pests and diseases are consistent with the daily management of other lavenders in the plantation.

In this study, the soil was sampled in April 2023 in the study area from four planting years in the same lavender garden in the unplanted (L0) lavender field where no lavender grows, one year of planting (L1), three years of planting (L3), and five years of planting (L5), respectively. Three plants with similar plant height and crown size were randomly selected from each of the four sampling points, with three replicates per sample point. Before collecting the samples, a spade was used to remove the debris from the ground, and then opened to dig the soil up to the roots care must be taken to avoid digging to break the roots, the depth of about 0–20 cm from the surface of the soil until the whole lavender plant was dug out, and the soil sticking to the roots was gently shaken off as the inter-root soil Three soil samples were collected from each sample site, the samples were put into sterilized bags and mixed thoroughly, and the samples were preserved in liquid nitrogen and immediately transported back to the laboratory, then the samples were placed on ice, and the impurities in the samples, such as debris, fallen leaves, plant and animal residues, were removed using tweezers, and the samples were thoroughly mixed and passed through a 2 mm steel sieve, and placed in 50 ml centrifugal tubes and stored in a refrigerator at -80°C.

### Soil physical and chemical characterizations

The pH meter was used to determine the soil pH [29]; soil organic carbon (TOC) content was determined by potassium dichromate oxidation method [30]; The Kjeldahl method was used to measure soil total nitrogen (TN) [31]; Soil available phosphorus content (AP) was measured by molybdenum-antimony antimony colorimetric method [32]; Soil total phosphorus (TP) and total potassium (TK) were measured by NaOH alkali fusion method; Soil and flame photometer method respectively [33].

### DNA extraction, PCR amplification and high-throughput sequencing

CTAB method for DNA extraction: 1000ul of CTAB, lysozyme and soil sample were added into 2ml centrifuge tube, inverted and mixed to make it fully lysed, then centrifuged at 65°C for 30 min, the supernatant was removed by centrifugation, and an equal volume of phenol (pH8.0): chloroform: isoamyl alcohol (25:24:1) was added and centrifuged at 12000 rpm for 10 minutes to remove the residual phenol. The supernatant was added and an equal volume of chloroform: isoamyl alcohol (24:1) was added. (24:1) is to remove the residual phenol, aspirate the supernatant to 1.5mL centrifuge tube, add isopropanol, placed in -20°C precipitation for 30 min, centrifugation 12000rpm 10 min, discard the supernatant, washed with 1ml of 75% ethanol for 2 times, dry at room temperature, add ddH_2_O to dissolve the DNA samples, and then add RNase A 1ul digestion of RNA, 37°C rest 15min [34]. DNA concentration was detected using a NanoDrop ND-2000 spectrophotometer (NaonoDrop Technologie, Wilmington, DE, USA). DNA quality was determined by 1% (w/v) agarose gel electrophoresis. Each final DNA sample was mixed with the same volume from triplicate sampl to avoid bias in sampling and extraction. All the DNA was stored at −20°C until further PCR amplification.

PCR amplification was performed using the universal fungal primers ITS5-1737F (5’-GGAAGTAAAAGTCGTAACAAGG-3’) and ITS2-2043R (5’-GCTGCGTTCTTCATCGATGC-3 ’). PCR reactions were carried out with 30μL of Phusion® High-Fidelity PCR Master Mix (New England Biolabs).PCR reaction system and program (30 μL): fusion masterbatch (2×) 15 μL, forward primer (1oul/μL) 1 μL(1oul), reverse primer (1oul/μL) 1 μL, (1oul) g DNA (1 ng/μL) 10 μL (10 ng) H_2_O_2_ μL; Reaction program: pre-denaturation at 98°C for 1 min; 30 cycles including (at 98°C for 10 s; annealing at 50°C for 30 s; and at 72°C for 30 s); the reaction was extended for 5 min at 72°C, and the reaction was stopped at 10°C. Aliquots were mixed according to the PCR product concentration, and the PCR products were purified by agarose gel electrophoresis using 1x TAE at 2% concentration after sufficient mixing, and the target bands were recovered using the Universal DNA (Tian Gen, China) recovery kit [35].

The library was constructed using the NEB Next ® Ultra DNA Library Prep Kit (Illumina, San Diego, CA, USA), and the constructed library was detected and quantified by Q-PCR using Agilent 5400 (Agilent Technologies Co Ltd., USA); The constructed libraries were checked for quality, and after the libraries were qualified, they were sequenced on-line using NovaSeq6000 (Illumina, San Diego, CA, USA). Sequencing of all the samples in this study was performed by Shenzhen Micromeritics Alliance Ltd (Shenzhen, China).

### Data processing

1. QIIME2 and R software (version 3.5.1) were used to analyze the α-diversity and β-diversity of the fungal communities and visualized by non-metric multidimensional scaling (NMDS) ordination, and box plots of diversity and dominant organisms were generated using the R language.
2. One-way analysis of variance (ANOVA) and multifactorial comparisons of the samples were performed using SPSS 27.0, and the Dunn test was used to compare the significance of the differences between samples in different groups. Lefse (linear discriminant analysis of effect size) was the taxon used to test the abundance of differences between the groups.
3. Correlation analysis between dominant fungi and soil physicochemical factors was carried out using SPSS, and redundancy analysis (RDA) of samples was used to analyze the relationship between fungal communities and environmental factors using the R language. Canoco5 software was used for mapping. Functional prediction was performed by FUN Guild.

## Results

### Analysis of soil physicochemical properties

Soil organic matter content is key to the survival of beneficial soil microorganisms and a decrease in organic matter also signals a decrease in beneficial microorganisms. Excessive acidity of soil often lead to acidification and increase the reproduction rate of some fungi, while excessive alkalinity will lead to alkalization of the soil, limiting crop growth. As shown in Table 1 soil pH, TOC, TN, TP, TK and SP varied significantly (*P*<0.05) with increasing planting years. As planting years increased, Soil pH first increased and then stabilized, while TOC, TN, and TP contents decreased, TK and SP contentsincreased. Data on the physical and chemical characteristics of soils samples are shown in S1 Table.

**Table 1:** Physical and chemical characteristics of soil samples.

	pH	TOC g/kg	TN mg/kg	TP mg/kg	TK mg/kg	SP mg/kg
**L0**	7.78±0.08c	5.62±0.01b	1.39±0.07a	1.14±0.04a	21.50±0.41c	0.07±0.00d
**L1**	8.44±0.08b	5.46±0.01b	1.36±0.06a	1.07±0.02b	21.92±0.02b	0.01±0.00c
**L3**	8.61±0.07a	5.46±0.21b	1.23±0.14b	0.97±0.01c	22.06±0.01b	0.10±0.01b
**L5**	8.58±0.08ba	4.67±0.12c	1.15±0.02c	0.82±0.01d	22.60±0.08a	0.13±0.00a

Note: Data were represented as mean ± SD. Different letters indicate significant differences among means (*P*<0.05).

### Diversity index and richness of the fungal community

**Fig 1:**
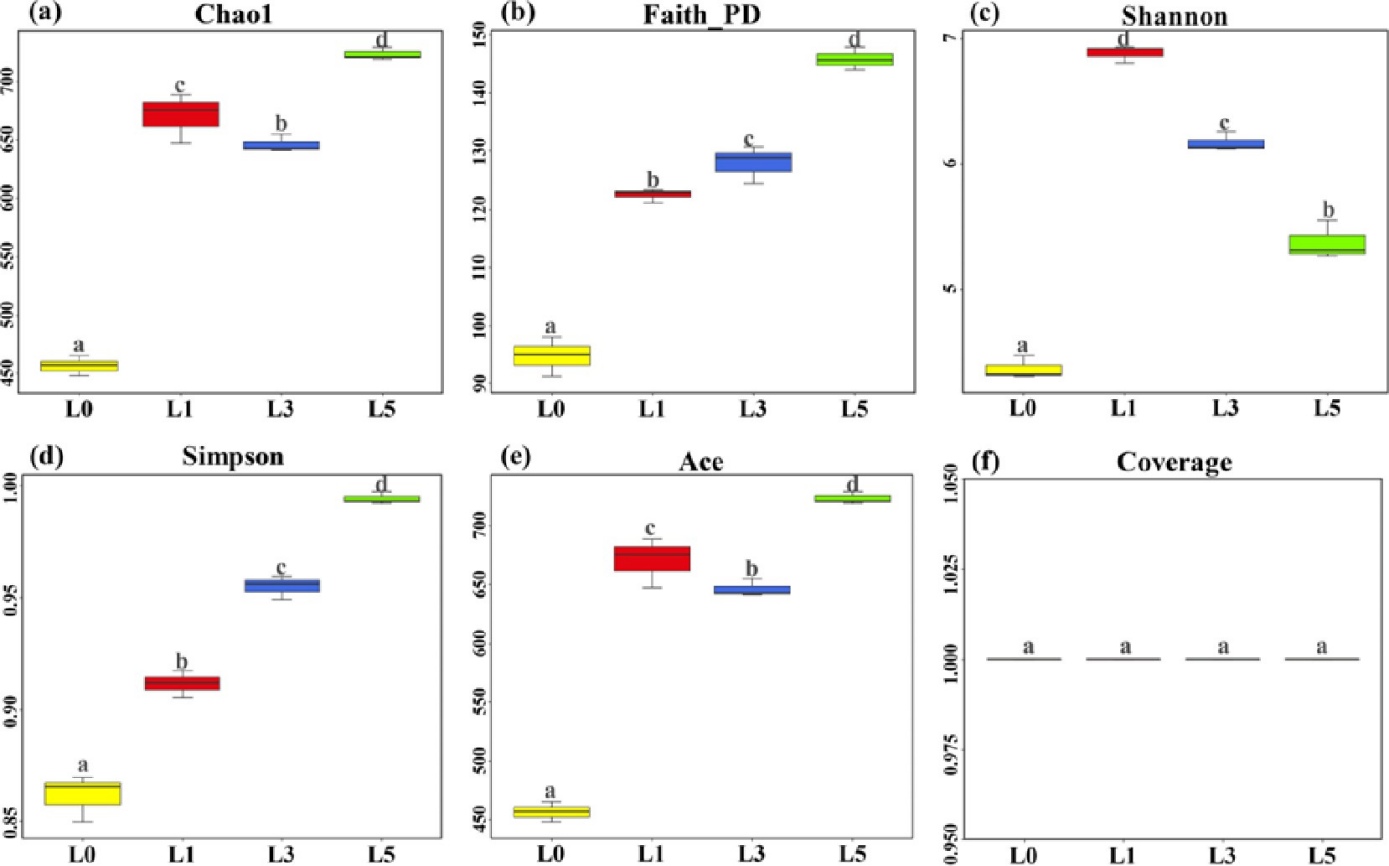
Alpha diversity analysis of fungal communities. Note: L0, L1, L3, and L5 represent 0, 1, 3, and 5 years of continuous cropping, respectively. Significance between different groups was compared using Duncan, smultiple range test (*P* < 0.05; n = 3).

By sequencing the V5 region of the fungal 18S rDNA gene in lavender soil samples, the number of original sequences obtained was 1,922,679, and after filtering out the low-quality sequences, the total number of valid sequences obtained was 1,717,299, and the quantity of operational taxonomic units, or OTUs, in the samples from each of the four categories of L0, L1, L3 and L5 were 604, 1,262, 912 and 1,018, accounting for 56.93%, 66.25%, 60.64% and 63.63% of total ASVs(Amplicon sequence variant), respectively, which indicated that the endemic species in the soil increased dramatically in L1. Compared with L0, L1, L3 and L5 had higher proportions of 68.61%, 62.77% and 66.25% of the total number of ASVs in each group, respectively. As shown in Fig 1. the order of Simpson’s index was L5<L3<L1<L0. An increase in Simpson’s index indicated a decreasing trend in fungal community diversity. The order of Chao1 index and ACE index was L5<L1<L3<L0, indicating that species abundance and richness showed a decreasing trend followed by an increasing trend. The order of Faith pd index, L5<L3<L1<L0, indicated a decreasing trend in species abundance and richness. L1<L0, indicating a significant increase in phylogenetic diversity. The coverage rate was 100%, indicating that the samples were sufficiently collected and most of the fungal species in the lavender rhizosphere were detected. Data on the alpha-summary are shown in S2 Table.

**Fig 2:**
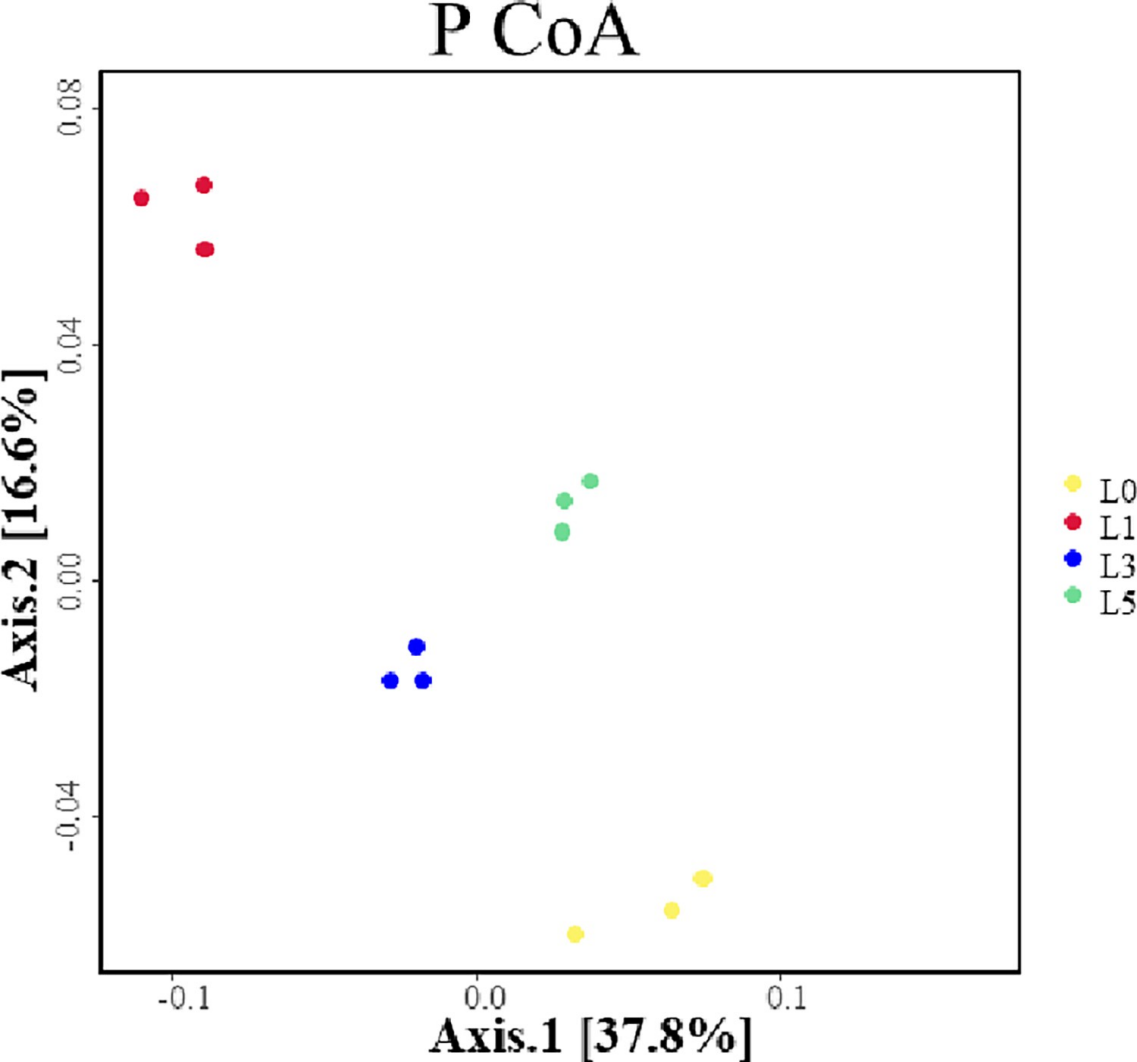
Two-dimensional sorting plot of PCoA analyzed samples.

As shown in Fig 2, The results of P CoA analyses showed that the soil fungal community structure of lavender varied greatly among the four planting years., and an explanation of the first and second main components acccounted for 37.8% and 16.6% of the fungal community structure, respectively, with a cumulative explanation of 54.4%. β-diversity analysis revealed that there were variations between the four treatment groups, between the two and between the two, of which the L5 fungal community differed the most from that of the L0 fungi and from that of the L1 fungal community. The largest difference indicated that the composition of the soil fungal population in the lavender rhizosphere was more influenced by the planting year, and this effect increased with increaseing year of planting. Data on the PCoA are shown in S3 Table.

### Composition and structure of the fungal community

A total of, 607 species from 32 phyla, 64 classes, 122 orders, 234 families, and 434 genera were found in the fungal community between the roots of lavender plants. The relative abundance of dominant bacteria in all four planting years changed significantly as the number of planting years increased.

**Fig 3:**
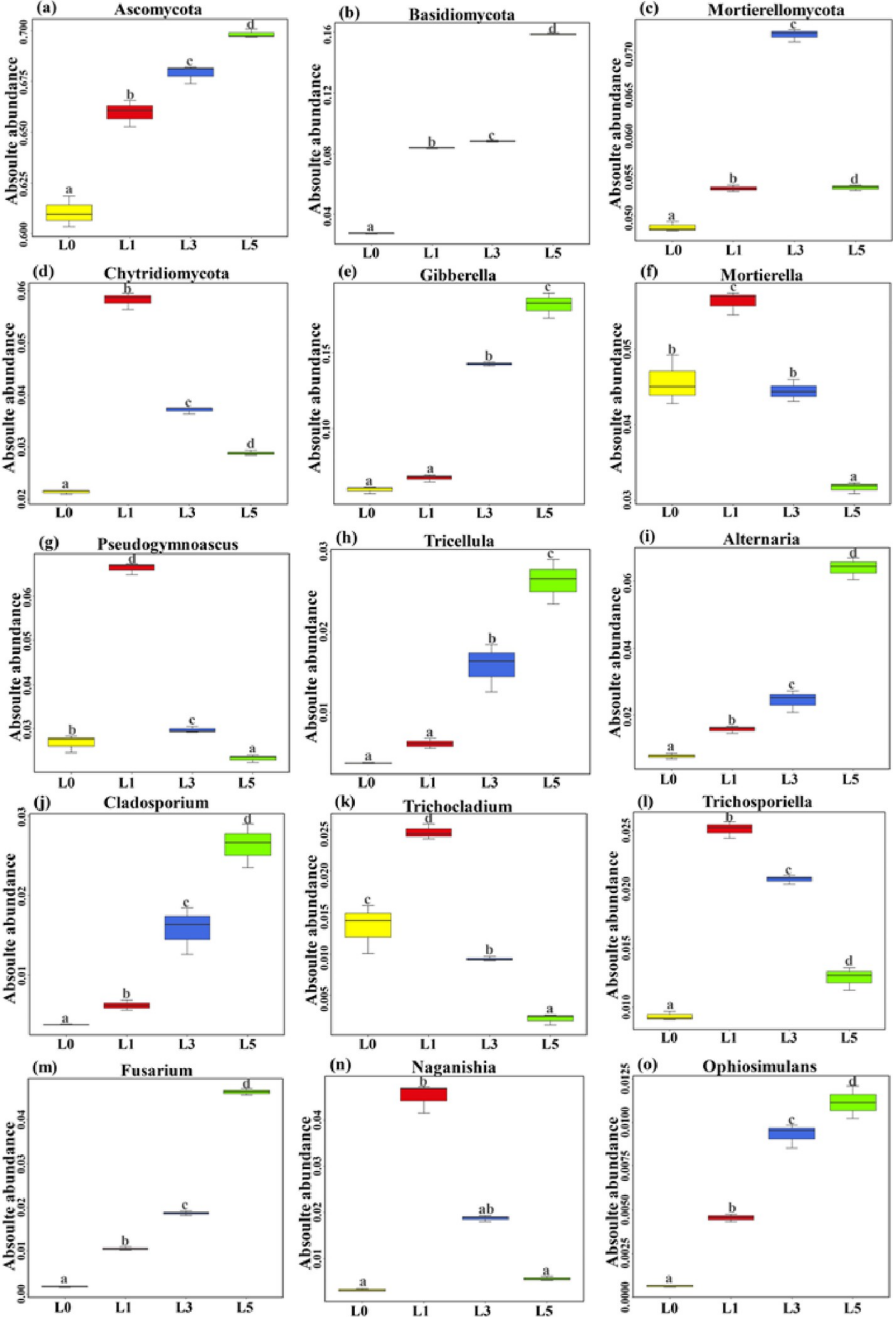
Absolute abundance of dominant fungal composition. **Note:** Absolute abundances of the dominant fungal composition across all samples. L0, L1, L3, and L5 represent 0, 1, 3, and 5 years of continuous cropping, respectively. Different letters indicate significant differences (Duncan, smultiple range test, *P* < 0.05).

As shown in Fig 3, at the phylum level, a total of 32 phyla were detected, of which 8.75% were unclassified, and those with a relative abundance greater than 5% were dominant phyla, which were Ascomycota, Basidiomycota, Mortierellomycota, and Chytridiomycota, respectively. In the planted lavender soil Ascomycota, Basidiomycota gradually increased with the planting years being extended, the relative abundance of Ascomycota was 65.96%, 67.91% and 69.83% in order; the relative abundance of Basidiomycota was 8.38%, 8.83% and 15.86% in order; Initially, the relative abundance of Mortierellomycota declined, followed by an increase; with the extension of planting years, its relative abundance first decreased and then increased. The relative abundance of Mortierellomycota was 5.34%, 7.21% and 5.35%, and the relative abundance of Chytridiomycota decreased with the increase of planting years, which were 5.83%, 3.72% and 2.89% in the order of planting years. Data on the phylum are shown in S4 Table.

As shown in Fig 3, at the genus level, the proportional abundance of *Gibberella*, *Mortierella*, *Pseudogymnoascus*, *Tricellula*, *Alternaria*, *Cladosporium*, *Trichocladium*, *Trichosporiella* was greater than 1%, while *Fusarium*, *Naganishia*, *Ophiosimulans*, *Monilia*, and 12 other genera, and unknown fungi accounted for 40.96%-70.58%. Among them,*Gibberella*, *Cladosporium*, *Alternaria*, *Fusarium*, *Monilia*, and *Tricellula* were all harmful fungi; *Mortierella*, *Pseudogymnoascus*, *Trichocladium(mycorrhizal fungi)*, *Trichosporiella (mycorrhizal fungi)*, *Naganishia (mycorrhizal fungi)* are all beneficial fungi, *of* which the role of *Ophiosimulans* varies depending on the plants, either pathogenic or mycorrhizal. *Ophiosimulans* can be either pathogenic or mycorrhizal. Data on the genus are shown in S5 Table.

**Fig 4:**
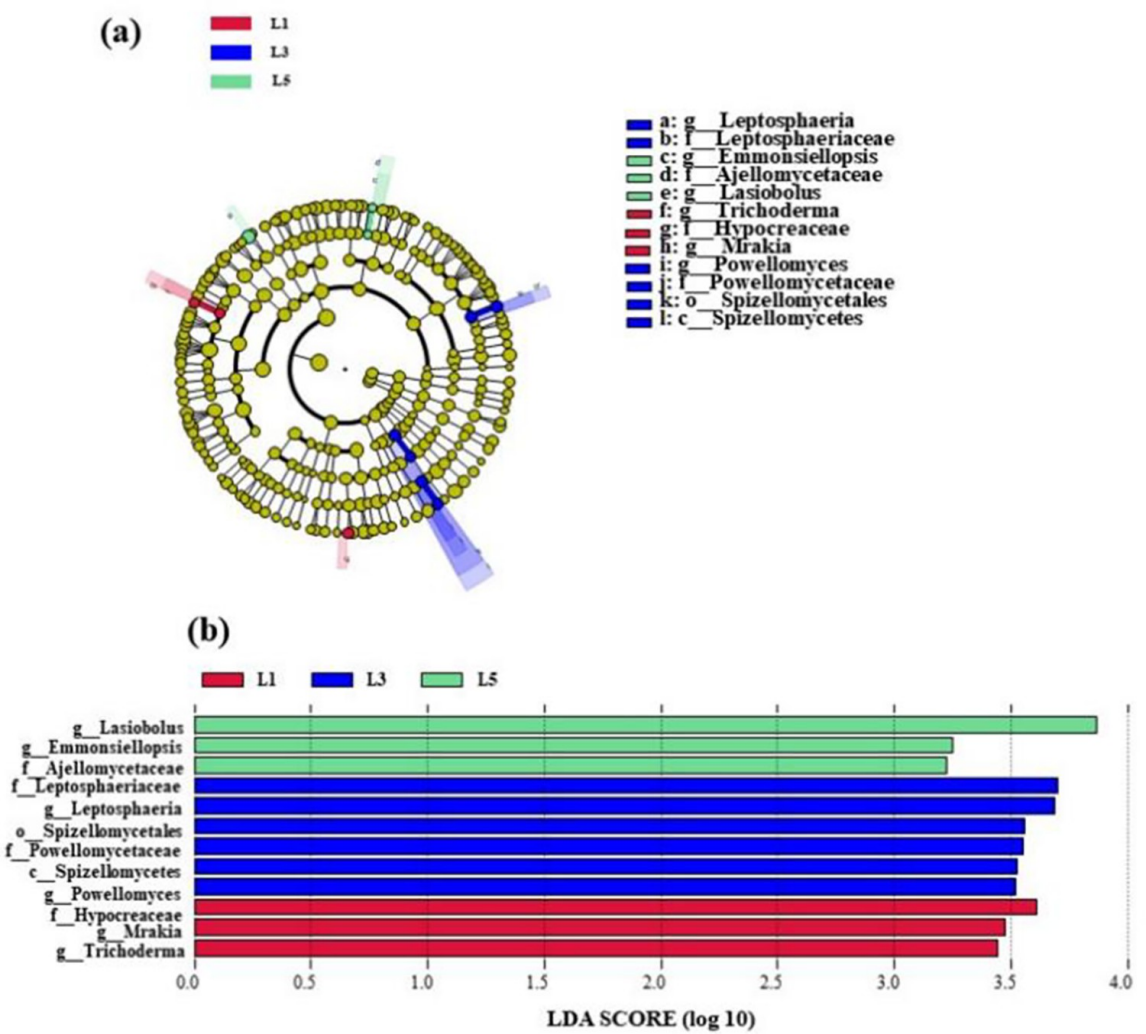
Results of flora statistics (a): Evolutionary branching diagram (b): LDA score.

Fungal taxa associated with the four planting years were identified by linear discriminant analysis (Lef Se). Fungal marker identification was utilized to differentiate taxa across the four planting years. The taxa displayed only meet the linear discriminant analysis (LDA) significance level of >2.0(Fig 4). Of these, no marker species were present in L0 soils, and the main divergent species in L1 were *Trichoderma*, *Mrakia*, and *Hypocreaceae*; the main divergent species in L3 were *Powellomyces*, *Spizellomycetes*, *Powellomycetaceae*, *Spizellomycetales*, *Spizellomycetaceae*, *Leptosphaeria*, and *Leptosphaeriaceae*; the main divergent species in L5 were *Lasiobolus*, *Emmonsiellopsis*, and *Ajellomycetaceae*. where *Trichoderma*, *Mrakia*, *Powellomyces*, *Spizellomycetes*, *Powellomycetaceae*, *Spizellomycetales*, *Leptosphaeria*, *Leptosphaeriaceae* showed an increase and then a decrease with the number of years of planting, *Hypocreaceae* and *Lasiobolus* showed a decrease and then an increase *Emmonsiellopsis* and *Ajellomycetaceae* gradually increased. Results of flora statistics are as shown in S6–S8 Tables.

### Correlation analysis between the diversity index of lavender rhizosphere fungi and soil factors

There is a certain correlation between the diversity index of lavender rhizosphere fungi and the physicochemical properties of the soil. As shown in Table 2, the Chao1 index, Simpson index, and Faith-pd index of lavender fungi are significantly positively correlated with pH, total potassium, and available phosphorus (*P* < 0.05); the Shannon index is significantly positively correlated with pH (*P* < 0.05); the Chao1 index, Simpson index, and Faith-pd index of lavender fungi are significantly negatively correlated with total nitrogen, total phosphorus, and organic carbon (*P* < 0.05). The diversity index of lavender rhizosphere fungi shows a significant negative correlation with total nitrogen, total phosphorus, and organic carbon. Therefore, the soil factors that have a more significant impact on the diversity and abundance of lavender rhizosphere fungi are pH, total potassium, and available phosphorus.

**Table 2:** Correlation analysis between rhizosphere fungi diversity index of lavender and soil factors.

diversity index	pH	TN	TP	TK	TOC	SP
**chao1 index**	0.921**	-0.705*	-0.784**	0.804**	-0.647*	0.773**
**Simpson index**	0.862**	-0.922**	-0.940**	0.853**	-0.793**	0.929**
**Shannon index**	0.712**	-0.082	-0.122	0.236	0.030	0.092
**Faith -pd index**	0.895**	-0.845**	-0.924**	0.920**	-0.815**	0.909**

Note: Positive numbers represent positive correlation, negative numbers represent negative correlation, and

** represents significance (*P* < 0.01).

### Correlation analysis between the community structure of lavender rhizosphere fungi and soil factors

**Fig 5:**
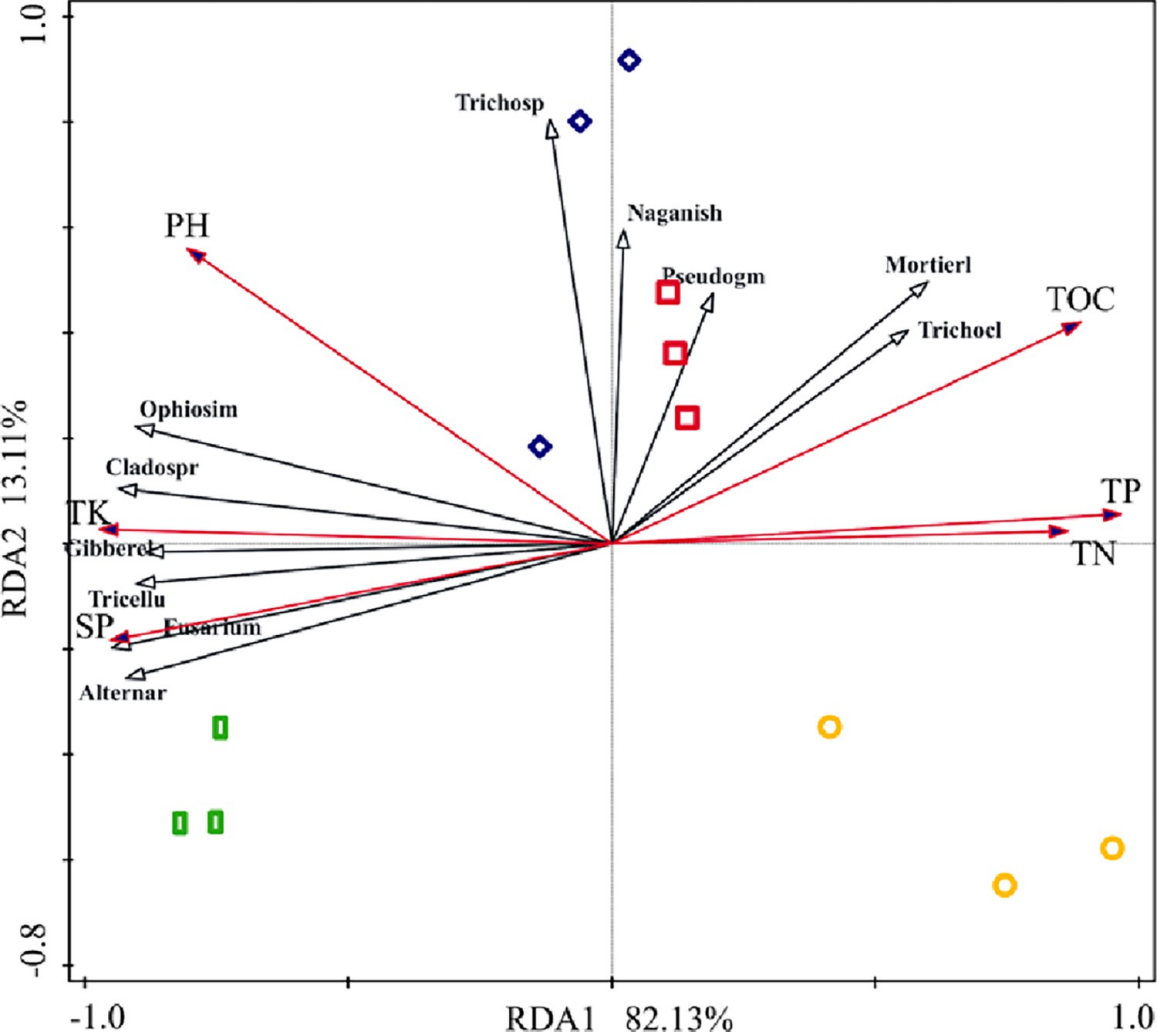
Redundancy analysis of soil fungal communities and soil physical and chemical properties.

Redundancy analysis as shown in Fig 5, where the black solid line represents dominant fungal genera and the red solid line represents six physicochemical factors. RDA axis 1 explains 82.13% of the variance, while axis 2 explains 13.11% of the variance, with a cumulative explanatory rate of 95.24%. Among these factors, pH, total potassium, and available phosphorus had the greatest impact on the fungal community. Correlation analysis between dominant fungal genera and soil physicochemical properties revealed (Table 3) significant positive correlation (*P* < 0.05) between the relative abundance of *Gibberella* and pH, and highly significant positive correlations (*P* < 0.01) with total nitrogen, total phosphorus, total potassium, organic carbon, and available phosphorus. *Mortierella* showed highly significant positive correlations (*P* < 0.01) with total nitrogen, total phosphorus, and organic carbon, and a highly significant negative correlation (*P* < 0.01) with available phosphorus. *Tricellula* and Alternaria exhibited highly significant positive correlations (*P* < 0.01) with total potassium and available phosphorus, and highly significant negative correlations (*P* < 0.01) with total nitrogen, total phosphorus, and organic carbon. *Cladosporium* displayed highly significant positive correlations (*P* < 0.01) with pH, total potassium, and available phosphorus, and highly significant negative correlations (*P* < 0.01) with total nitrogen, total phosphorus, and organic carbon. *Trichocladium* showed highly significant positive correlations (*P* < 0.01) with total phosphorus and total potassium, and highly significant positive correlation (*P <* 0.01) with available phosphorus. *Fusarium* demonstrated highly significant positive correlations (*P* < 0.01) with organic carbon and available phosphorus, and highly significant negative correlations (*P* < 0.01) with total nitrogen, total phosphorus, and total potassium. *Ophiosimulans* exhibited highly significant positive correlations (*P* < 0.01) with pH, organic carbon, and available phosphorus, and highly significant negative correlations (*P* < 0.01) with total nitrogen, total phosphorus, and total potassium.

**Table 3:** Correlation analysis between dominant bacterial flora and soil physical and chemical.

dominant fungal genera	pH	**	TP	TK	TOC		SP
***Gibberella***	0.716*	-0.954**	-0.962**	0.830**	-0.802**	0.930**	
***Mortierella***	-0.207	0.763**	0.745**	-0.546	0.740**	-0.773**	
***Pseudogymnoascus***	0.140	0.466	0.399	-0.181	0.339	-0.381	
***Tricellula***	0.691*	-0.925**	-0.959**	0.855**	-0.837**	0.946**	
***Alternaria***	0.603*	-0.875**	-0.943**	0.879**	-0.930**	0.960**	
***Cladosporium***	0.827**	-0.932**	-0.952**	0.888**	-0.800**	0.933**	
***Trichocladium***	-0.218	0.658*	0.801**	0.731**	-0.518	-0.714**	
***Trichosporiella***	0.576	0.202	0.056	0.021	0.114	-0.710	
***Fusarium***	0.669*	-0.940**	-0.898**	-0.966**	0.894**	0.988**	
***Naganishia***	0.350	0.234	0.172	0.199	-0.031	-0.176	
***Ophiosimulans***	0.867**	-0.721**	-0.918**	-0.928**	0.858**	0.897**	

Note: Positive numbers represent positive correlation, negative numbers represent negative correlation,

* Representative significant correlation (*P* < 0.05)and

** Highly significant correlation (*P* < 0.01).

### Functional groups of fungi

FUN Guild functional prediction was used to analyze the functional classifications of fungi in soil samples and the abundance of each functional classification in different samples. Results showed that the relative abundance of 15 taxa, including Plant Pathogen and Plant Saprotroph, in the inter-root zone of lavender in the four planting years was relatively high, as shown in Table 4. Among them, Plant Pathogen, Plant Saprotroph, Animal Pathogen, Fungal Parasite, Leaf Saprotroph and Lichen Parasite had significant variations (*P* < 0.05). Six species had significant variation (*P* < 0.05). Animal Pathogen decreased significantly with increasing planting years; Fungal Parasite, Leaf Saprotroph and Lichen Parasite increased significantly and then decreased significantly; Plant Pathogen (Plant Parasite) Plant Pathogen and Plant Saprotroph were significantly increased. the highest Plant Pathogen was found in the soil of L5, which made the plant to have early warning of pests and diseases. Functional predictions and abundance are as shown in S9 Table.

**Table 4:** Functional predictions and abundance top 15.

Taxon	L0	L1	L3	L5
**Animal Pathogen**	0.12±0.01b	0.11±0.04ab	0.09±0.01ab	0.07±0.01a
**Bryophyte Parasite**	0.00±0.00a	0.00±0.00a	0.01±0.01a	0.00±0.00a
**Dung Saprotroph**	0.01±0.01a	0.03±0.03a	0.01±0.00a	0.02±0.03a
**Ectomycorrhizal**	0.01±0.09a	0.03±0.02a	0.01±0.01a	0.02±0.02a
**Endomycorrhizal**	0.00±0.00a	0.00±0.00a	0.00±0.00a	0.00±0.00a
**Endophyte**	0.12±0.02a	0.09±0.02a	0.01±0.00a	0.01±0.01a
**Epiphyte**	0.00±0.00a	0.00±0.00a	0.00±0.00a	0.00±0.00a
**Ericoid Mycorrhizal**	0.00±0.00a	0. 15±0.00a	0.01±0.01a	0.00±0.00a
**Fungal Parasite**	0.08±0.03b	0.05±0.01ab	0.05±0.01ab	0.04±0.02a
**Leaf Saprotroph**	0.00±0.00a	0.00±0.00b	0.00±0. 004a	0.00±0.00a
**Lichen Parasite**	0.07±0.03b	0.04±0.01ab	0.05±0.01ab	0.04±0.01a
**Litter Saprotroph**	0.02±0.01b	0.03±0.01ab	0.03±0.01ab	0.04±0.02a
**Plant Pathogen**	0.22±0.06a	0.16±0.02a	0.23±0.03a	0.19±0.08a
**Plant Saprotroph**	0.01±0.00a	0.03±0.02ab	0.05±0.02b	0.04±0.02b
**Soil Saprotroph**	0.06±0.03a	0.10±0.02a	0.0536±0.00a	0.08±0.04a

## Discussion

### Relationship between physicochemical properties and Lavender rhizosphere fungi

In this study, pH values increased and then leveled off with the increasing years of planting, suggesting a gradual alkalization of soil. Lavender will be subjected to alkaline soil stress, with soil pH strongly correlated with most of the major fungal genera [36,37]. Phosphorus helps increase carbon sequestration and water use efficiency, thus affects plant growth. High soil phosphorus levels usually result in lower AMF colonization, which reduces beneficial mycorrhizae and the ability to use their own resistance against pathogens [38–40]. Total nitrogen tends to decrease, however, lower nitrogen levels reduce chlorophyll content and the number of leaf cells resulting in lower photosynthetic rates [38]. Potassium increases plant resistance to diseases and stresses, and the significant increase in total potassium indicated the activation of stress tolerance mechanisms in lavender [41].

### Fungal diversity and community variability

Diversity in the soil microbial community is essential for maintaining plant biodiversity, soil health and productivity [42–44]. Fungi are an important component of soil biomass. In addition to playing key roles in nutrient cycling and biological interactions, they are intimately involved in soil structure dynamics [45]. The number of planting years affected the diversity and richness of the rhizosphere fungal communities, and the soil fungal ASV increased and then decreased during successive plantings of lavender, Additionally,since the fungal communities in our study were influenced by the qualities of the soil, we postulated that soil fertility would increase fungal variety in the short term and decrease the number of soil fungal species over time, which was the same as in the study of Wu et al and different from that in the study of Dong et al. due to crop differences [46–48]. However, the accumulation and long-term ecological effects of homogeneous substances secreted by the root system of similar plants regulate the shifting of soil microbial populations and their structural disruption [49]. Successive monoculture barriers are usually ascribed to the deterioration of soil physicochemical properties and the accumulation of soil-borne pathogens or self-toxic chemicals [50–52]. Several investigations have demonstrated that modifications in the soil microbiota, including the growth of bacterial and fungal pathogens, are the main reason for the obstacles to continuous monoculture [53,54]. Therefore, continuous cultivation of lavender might cause the a decrease in the resistance of rhizosphere fungi to the soil micro-environment andto outside, thus limiting sustainable soil development.

In this study, the highest fungal community abundance was found in L1, and showed decreasing trend after L1, and most of the dominant fungi in L1 were beneficial fungi, while after L1, there was a trend toward a decline in the richness of the fungal community. This indicated that the soil decreased during successive planting of lavender, and this phenomenon also occurred in monoculture of Panax notoginseng, tea and peanut [48,54,55]. The relative abundance of pathogenic fungal diversity in both rhizosphere and rhizosphere soil of L3 was higher than that of L1. This may be due to the accumulation of phenolic compounds in the soil as the growing season increases, which stimulates the growth of pathogenic fungi which cause root rot [46,56,57]. In treatment L5, the pathogenic fungi continued to increase, but there was also colonization of AMF such as *Trichocladium*, *Trichosporiella*, and *Naganishia*, which can improve plant resistance, nitrogen fixation, and phosphorus solubilization, whereas the pathogenic fungi can inhibit plant growth [58].

Ascomycetes, Stramonium and Tephritobacterium were the most dominant phyla in this study, and the absolute abundance of Ascomycetes increasing with the increase of planting years, which was different from that of sweet potato and cucumber [59,60],but the same as that of Rhizome Coptidis [61], indicating that crop types have a different influence on dominant phyla richness. Ascomycetes phyla can form AMF, which is beneficial for crop growth, but there are also ascomycetes that can cause plant diseases resulting in reduced yields of cash crops [62]. The absolute abundance of ascomycetes increased, which is consistent with the results of the study on Chuanxin Lian [63],and this may be related to its participation in the decomposition of apoplastic material [64]. Mortierellomycota is crucial to the decomposition of soil organic matter, and its abundance may decline and have an impact on soil fertility.

Among the top 50 genera, some potentially pathogenic genera such as *Gibberella*, *Cladosporium*, *Alternaria*, and others are gradually increasing in abundance in the rhizosphere soil of lavender, and the increase of pathogenic fungi will most likely lead to lavender diseases. This is consistent with research showing that potential soilborne pathogens, and it often causes wheat scab and maize blast disease [65,66]. *Cladosporium* is a very common grapevine disease in Chile [67]. Black rot caused by *Alternaria arbusti* severely affects leaves and fleshy roots of carrot [68], and apple brown rot caused by *Alternaria arbusti* is the most prevalent post-harvest disease in all apple-producing regions [69]. Leaf spot disease of rubber trees is caused by *Bipolaris heveae* [70]. *Curvularia inaequalis* causes leaf blight symptoms on knotweed, bent grass, and bermudagrass [71], *Fusarium culmorum* is an important pathogen of wheat causing seedling blight, foot rot and head blight (*Fusarium head blight (FHB) or black star*) [72], *Fusarium biseptatum* infection of chickpea induces leaf yellowing and necrosis including upward progression from the stem base, and premature plant senescence [73]. Blackleg is caused by Ilyonectria liriodendri and Dactylonectria macrodidyma, which are capable of infecting grapevines through injured roots or the basal end of calluses [74]. These pathogens, which cause disease in other crops, are likely to have a potential effect on lavender growth and prevention of fungal diseases should be started after five years of lavender planting.

### Effects of continuous planting on ecological functions

With the extension of planting years, the accumulation of phytopathogenic and phytophthora flora and the gradual decrease of beneficial bacteria indicated that t the role of the fungal community gradually gave way to that of phytopathogenic and phytophthora. This is consistent with the findings indicating that the possible soilborne pathogens *Gibberella*, *Cladosporium*, *Alternaria*, and *Fusarium* were absolutely dominant genera in L3 and L5 soils. This study only analyzed the changes in the soil fungal community of lavender and its relationship with environmental factors in the four planting years, and did not specifically investigate the relationship between the occurrence of lavender diseases and the soil fungal community in the four planting years. Therefore, the next step is to further investigate the relationship between different planting years and soil properties, soil microbial communities and disease occurrence, in order to reveal the mechanism of the occurrence of continuous crop disorder in lavender.

### Mechanisms of inter-root microbial-plant interactions

At the molecular level, the interaction between plants and inter-root microorganisms is realized through the reciprocal communication of signaling molecules; some compounds in plant root secretions can act as signaling molecules to attract specific types of microorganisms to symbiose with them [75]. Plants produce and release multiple species of compounds, such as rhizoids, hormones, and secondary metabolites, of which rhizoids are able to stimulate the colonization and growth of beneficial microorganisms [76]. Microorganisms also release signaling molecules to regulate plant growth and development. The interaction of such signaling molecules can elicit physiological responses in plants [77], such as growth hormone synthesis and antioxidant production in plants. Inter-root microorganisms can activate plant defense mechanisms against pathogen attack [78]. Plants can sense beneficial microorganisms by recognizing common microbial compounds produced by them, such as flagellin, lipopolysaccharides, extracellular polysaccharides, and chitin [79], and some beneficial microorganisms can produce induced systemic resistance (ISR) responses in plants as well as systemic acquired resistance (SAR) through the release of fungal-produced signaling molecules such as N-propionic acid [80,81]. These signaling molecules can trigger changes in gene expression in plants, leading to the activation of disease resistance-related genes and the production of defense substances that enhance plant resistance to pathogens [82]. Interactions between inter-root microbes and plants also involve a complex network of gene regulation [83]. In this process, plants respond to the presence and activities of inter-root microorganisms through molecular mechanisms such as transcription factors and signaling pathways. This response mechanism involves a series of changes in gene expression and physiological metabolism that further affect plant growth and development [84].

### Effects of root secretions on inter-root microorganisms

Root secretions are rich in organic substances, such as organic acids and amino acids, which provide energy and carbon sources for inter-root microorganisms. The composition of the substances secreted by different plants varies, affecting the diversity and function of the inter-root microorganisms. Pine root secretions, such as sucrose, fructose, and organic acids, can be metabolized by soil fungi to produce energy and regulate the activity and abundance of Aspergillus spp, Actinobacteria phylum, and Thick-walled fungi phylum [85]. Plants regulate the behavior of inter-root microorganisms by secreting phytohormones and other signaling molecules. This signaling communication can affect microbial growth, activity and metabolism, which in turn regulates plant-microbe interactions. Organic acids and sugars are believed to make up the majority of the root secretions from different plants and play a key role in the recruitment and regulation of root-associated microbial taxa in soil [86]. In the soil environments, microbial communities are often influenced by host plant root secretions that attract or repel free-living pathogenic bacteria [87], and studies have shown that plants utilize root secretions to regulate the surrounding soil environments, to shape the microbiota [88], and to regulate plant growth and development [89]. Zhou etal. [90] found that intercropping systems are more effective in regulating root growth and development buy usingroot secretions (potato) to alter the recruitment of inter-root microbiota through signaling chemicals released from the root secretions, thus improving the adaptability of intercropped plants (tomato).Luo [91] showed that when Panax pseudoginseng was infested with foliar pathogens above ground, the enhanced secretion of organic acids, sugars and amino acids in its root secretions inhibited soil-borne pathogens, enriched beneficial microorganisms and reduced soil-borne diseases below-ground.

### Effects of inter-root microorganisms on root secretions

Soil microorganisms play a crucial role in the processes of intra-soil material and energy cycling, soil structure maintenance and soil micro-ecological balance Microorganisms promote the decomposition, conversion and synthesis of soluble substances such as organic acids, ammonia and nitrogen through the decomposition of organic matter in root secretions [92], which are mainly recruited from the environment [93]. With the decomposition of organic matter at differentstages, the various biochemical indicators of the soil are constantly changing, different microorganisms alternately dominate, independently or in cooperation with other microorganisms, decompose and transform organic materials, and promote the decomposition of organic matter The structure and composition of microbial communities are affected by root secretions, and substances secreted by different plants can shape the ecological niches of microorganisms in the soil, affecting the diversity and stability of microbial communities. Some inter-root microorganisms have antagonistic effects on plant pathogens and protect plants from diseases by secreting antibiotics and producing volatile organic compounds [94].

## Conclusions

After continuous cultivation of lavender, the physico chemical properties of the soil changed, the diversity of the fungal communities decreased, the abundance and richness of species decreased and then increased, and the phylogenetic diversity increased; the structure of the soil fungal community varied considerably. The increase in the concentration of harmful bacterial in the soil, especially some potentialil pathogenic bacteria, and the decrease in the content of beneficial bacteria may be one of the reasons for the emergence of the succession barrier in lavender cultivation, In order to further understand the relationship between lavender and the inter-root microorganisms, and for better cultivation and management and alleviation of the succession barrier in the production of lavender, We will continue monitor the population dynamics of soil microorganisms throughout each plant growth phase and across seasonal transitions, we will continue to monitor the changes in soil microorganisms in the soil in real time during each growth period and seasonal change.

## Supporting information

S1 Table Physical and chemical characteristics of soil. (DOCX)

S2 Table Alpha-summary. (DOCX)

S3 Table Category1 unweighted unifrac PCoA.ord. (DOCX)

S4 Table Phylum. (DOCX)

S5 Table Genus. (DOCX)

S6 Table Category1 genus lefse LDA2.lefseinput. (DOCX)

S7 Table Category1 genus lefse. (DOCX)

S8 Table Category1 genus lefse LDA2. (DOCX)

S9 Table Function. (DOCX)
