# Supplementary figures and images for: Analysis of rhizosphere fungal diversity in lavender at different planting years based on high-throughput sequencing technology
Source: PLoS One. 2024 Oct 3;19(10):e0310929. doi: 10.1371/journal.pone.0310929 (PMC11449376; doi:10.1371/journal.pone.0310929)

Table S1 Physical and chemical characteristics of soil


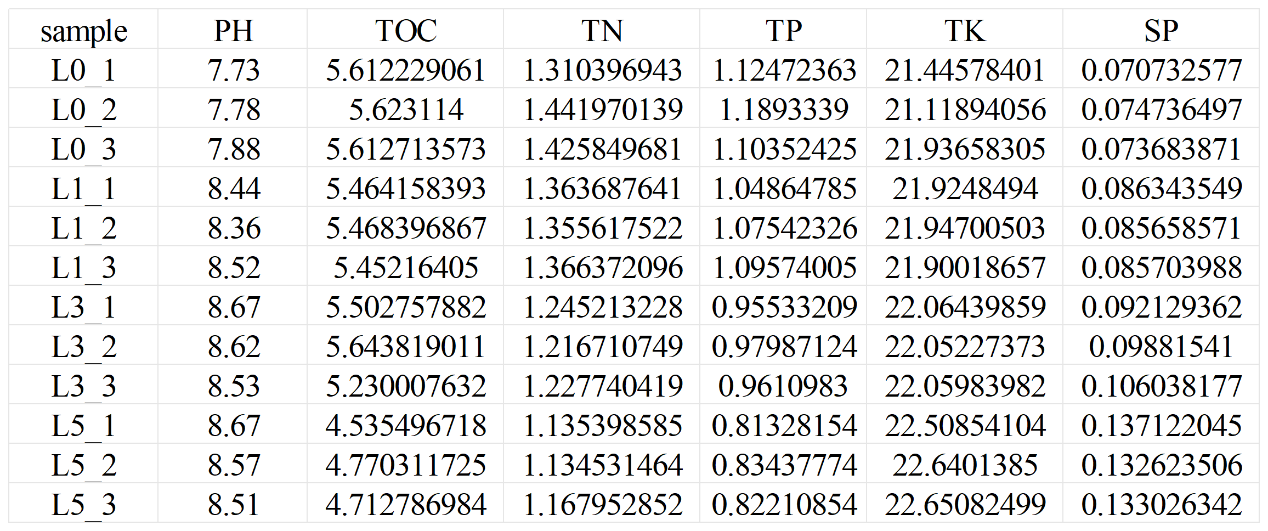

Supplement: S1 Table — (DOCX) [file pone.0310929.s001.docx]

Table S2 alpha-summary
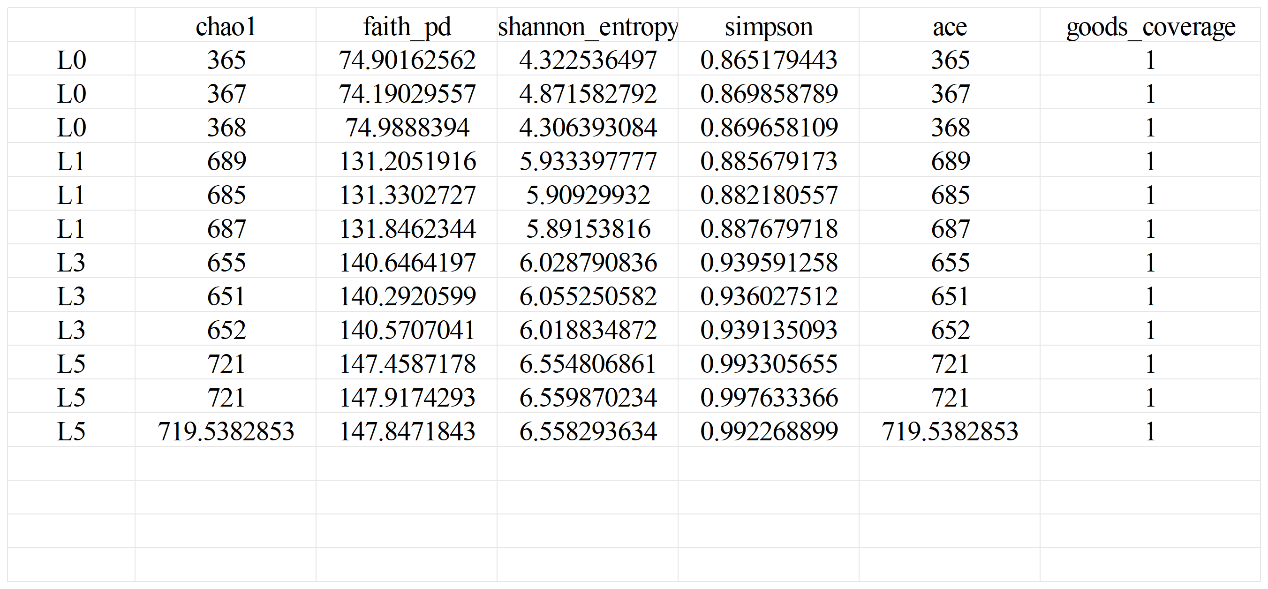

Supplement: S2 Table — (DOCX) [file pone.0310929.s002.docx]

Table S3 category1_unweighted_unifrac_PCoA.ord
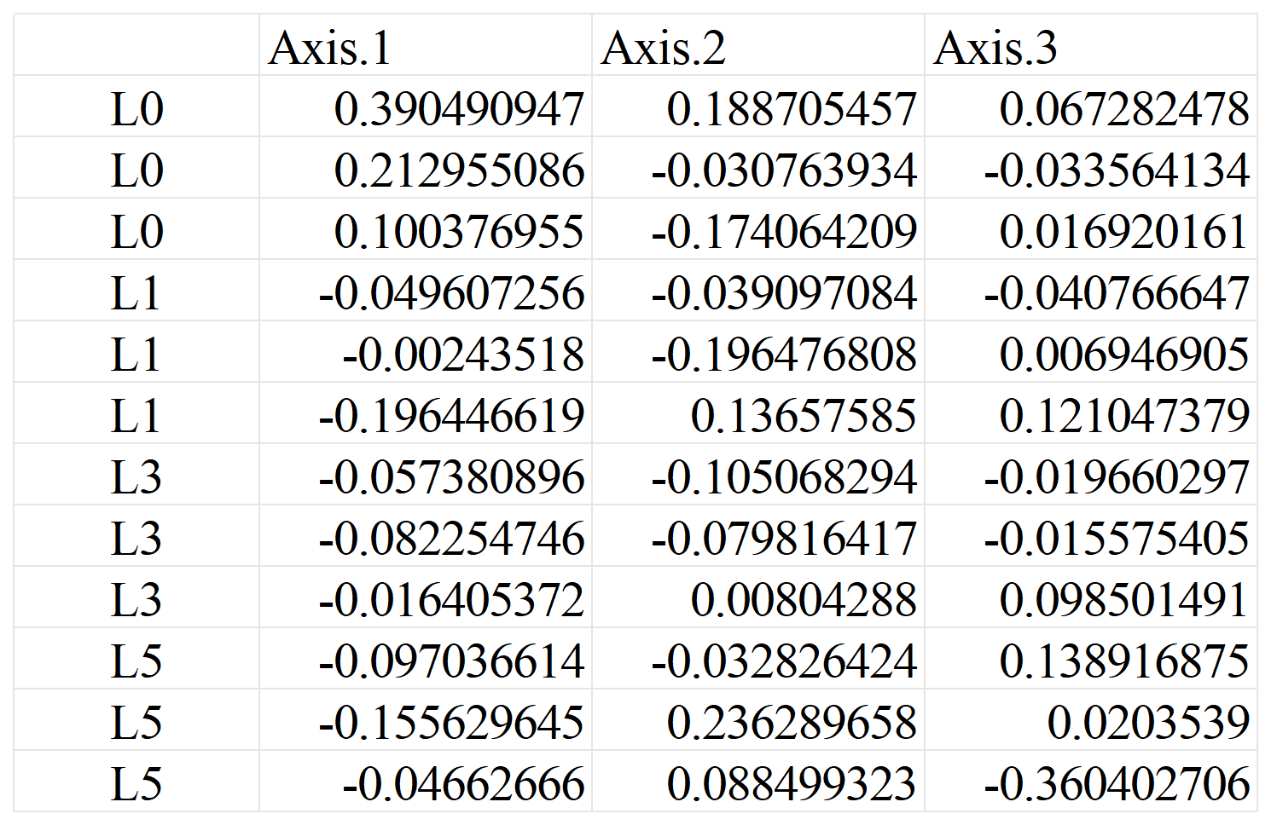

Supplement: S3 Table — (DOCX) [file pone.0310929.s003.docx]

Table S4 phylum
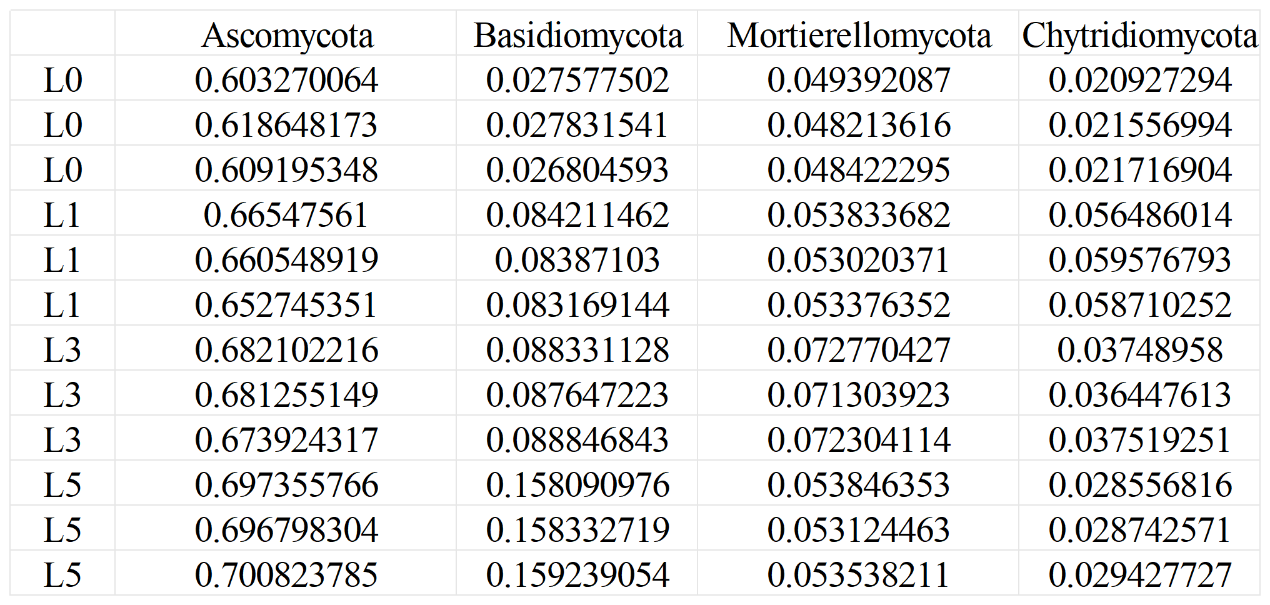

Supplement: S4 Table — (DOCX) [file pone.0310929.s004.docx]

Table S5 genus
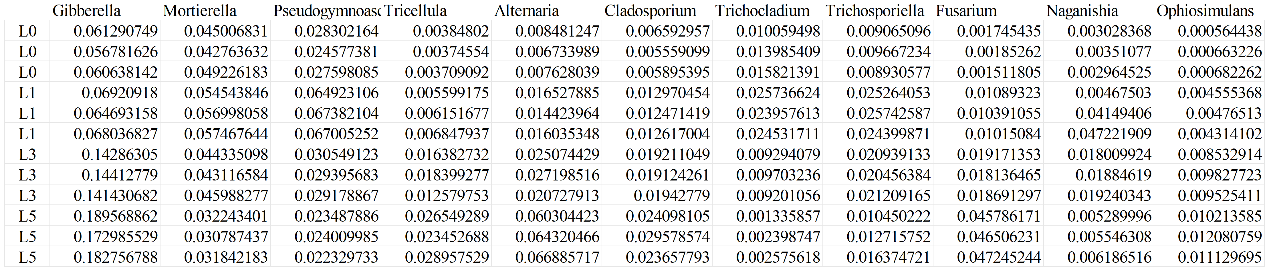

Supplement: S5 Table — (DOCX) [file pone.0310929.s005.docx]

Table S6 category1_Genus_lefse_LDA2.lefseinput


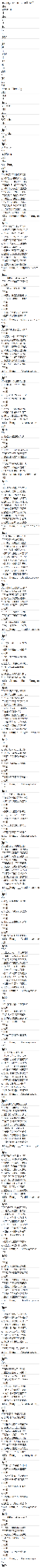

Supplement: S6 Table — (DOCX) [file pone.0310929.s006.docx]

Table S7 category1_Genus_lefse.
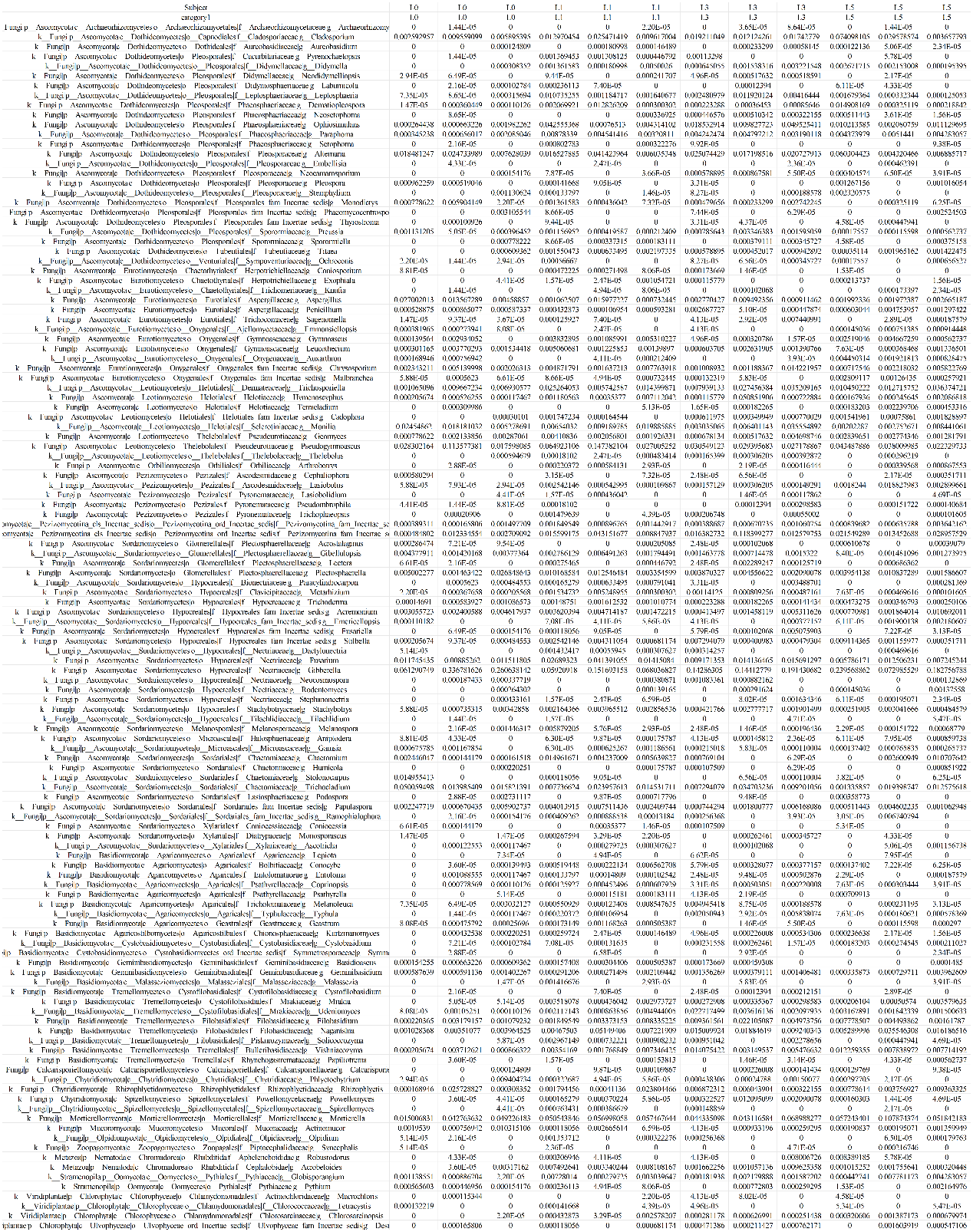

Supplement: S7 Table — (DOCX) [file pone.0310929.s007.docx]

Table S9 function.


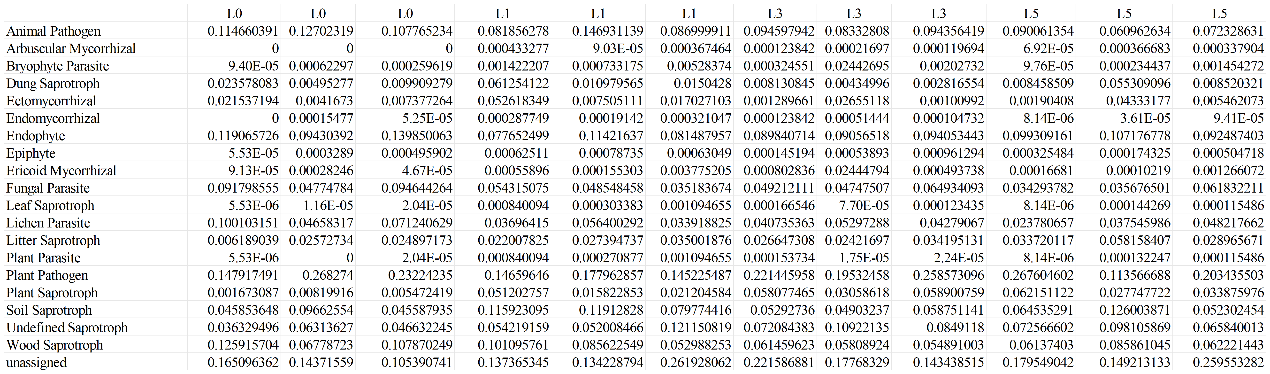

Supplement: S8 Table — (DOCX) [file pone.0310929.s008.docx]

Table S9 function.


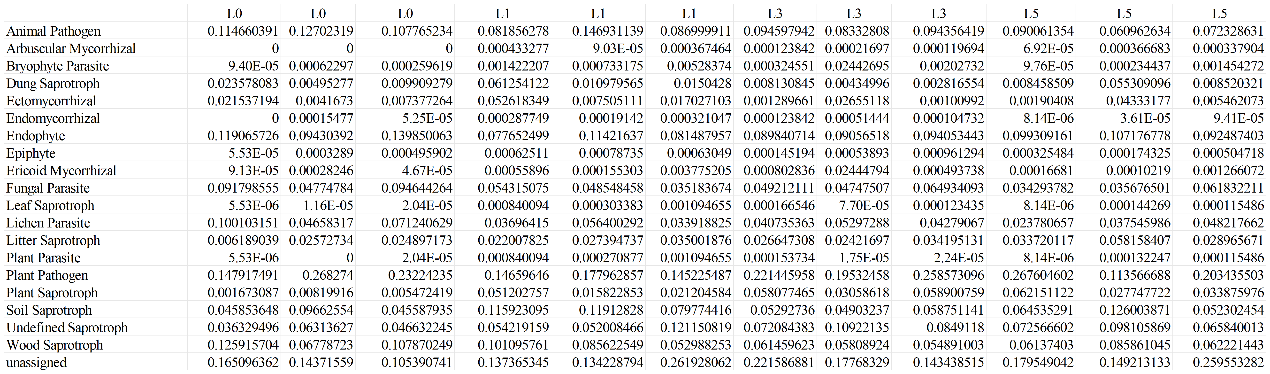

Supplement: S9 Table — (DOCX) [file pone.0310929.s009.docx]
